# Knowledge of the ovulatory cycle and its determinants among women of childbearing age in Haiti: a population-based study using the 2016/2017 Haitian Demographic Health Survey

**DOI:** 10.1186/s12905-022-02136-8

**Published:** 2023-01-02

**Authors:** David Jean Simon, Yasmeen Jamali, Comfort Z. Olorunsaiye, Jean-Marie Théodat

**Affiliations:** 1 Bureau d’Etudes et de Recherche en Statistiques Appliquées, Suivi et Evaluation (BERSA-SE), Port-au-Prince, Haiti; 2 Population Association of Pakistan (PAP), Islamabad, Pakistan; 3 Department of Public Health, Arcadia University, Glenside, PA USA; 4 Maître de Conférences, Paris 1 Panthéon Sorbonne University, Paris, France

**Keywords:** Ovulatory cycle, Knowledge, Determinants, Haiti, Gender, Development, Demographic and health survey

## Abstract

**Background:**

The knowledge of ovulatory cycle (KOC) is the base for natural family planning methods, yet few studies have paid attention to women’s KOC. This study aimed to assess the prevalence of correct KOC and its determinants among women of childbearing age in Haiti.

**Methods:**

Data from the nationally representative cross-sectional Haiti Demographic and Health Survey 2016/17 were used. STATA/SE version 14 was employed to analyse the data by computing descriptive statistics, Chi‑square, and binary logistic regression model to assess the socio-economic and demographic predictors of correct KOC. *P*-value less than 0.05 was taken as a significant association.

**Results:**

Out of 14,371 women of childbearing age who constituted our sample study, 24.1% (95% CI 23.4–24.8) had correct KOC. In addition, the findings showed that place of residence, respondent’s education level, wealth index, currently working, husband/partner’s education level, contraceptive use, exposure to mass media FP messages, and fieldworker visit were significantly associated with correct KOC.

**Conclusion:**

Policies should include increasing the literacy at community level as well as of individual women and their partners. Moreover, increasing awareness about family planning should be prioritized, especially for women from poor households and rural areas.

## Introduction

Ovulation in reproductive age women is a natural physiological process and an indicator of fertility [1]. The ovulatory cycle has three phases, egg formation (follicular), egg release (ovulation) and luteal. Ovulation is the most important event i.e., break-up of follicular and release of mature egg into the fallopian tube [2–4], it represents fertile window in the ovarian cycle [5]. The knowledge of the ovulatory cycle (KOC) is the basis of the natural family planning (NFP) methods [6]. Lack of correct KOC in sexually active women is likely to lead to unintended pregnancies in the absence of modern contraceptive use [7–10]. Thus correct KOC is important for women of reproductive age, both for planning of conception or avoiding unintended pregnancies and unsafe abortions [9, 11–13].

KOC has not received much attention in research on women’s reproductive health. However, recently, studies on the subject have reported that the prevalence of KOC among women is low. In India, only 11% of young women (age 15–24 years) had correct KOC [14]. Another study from Turkey on a young population also established that only 50% women had correct KOC and 40% of young people had correct KOC in the sample [15]. Similarly, studies from the global north reported low prevalence of adequate KOC among childbearing-age women [1, 16, 17]. Likewise, previous studies from Africa have shown low prevalence of adequate KOC among women; only 8.3% women of childbearing age in sub-Saharan Africa had correct knowledge of their fertile window, based on data from Demographic and Health surveys (2008–2017) [18], while this prevalence was estimated at 23.3% in Ethiopia [19].

Several studies have explored community and individual levels factors associated with correct KOC among women of childbearing age. These factors include women’s age, place of residence, region, religion, women’s education, husband/partner’s education, occupational status, wealth index, marital status, contraceptive use, menstruating in last 6 weeks, and being pregnant [18–24]. Further, existing studies revealed that exposure to mass media family planning messages and being visited by fieldworker are positively associated with women’s adequate KOC [18, 25–27].

Understanding the prevalence and determinants of correct KOC could help a country to overcome the problem. In the context of Haiti, a country characterized by a low prevalence of modern contraception use (23.4%), a high rate of unmet need for contraception (42.1%) and a large number of unwanted pregnancies (n = 413,000 in 2016) which are largely associated with socio-cultural, religious and economic factors [24, 25], no study to the best of the authors’ knowledge has focused on KOC level among women of reproductive age. In order to partially fill this gap, this paper aims to assess the prevalence of correct KOC and its determinants among women of childbearing age in Haiti.

## Materials and methods

### Study area and setting

Located between the Caribbean Sea and the North Atlantic Ocean, the Republic of Haiti has an area of 27,750 km^2^, and an estimated population of 11.4 million of which 60.5% are under 30 years old. Slightly more than half (50.5%) of people are women, 53.2% of whom are aged 15–49 (i.e. they are of childbearing age) [26]. Nearly 60% (58.4%) of the Haitian population lives in urban areas [27]. Moreover, Haiti is one of the poorest countries in Latin America and the Caribbean region: the poverty rate is estimated at 58.5% (40.6% in urban vs 74.9% in rural areas) [28].

### Data source

This present research used data from the most recent cross-sectional Haitian Demographic Health Survey (HDHS), a national level survey conducted over 5 months from November 2016 to April 2017, and implemented by the Haitian Institute for Children (HIC) in collaboration with the Haiti National Bureau of Statistics (HNBS), and the Ministry of Public Health and Population (MPHP). The survey gathered information on household population and characteristics, fertility, marriage, sexual activity, nutrition, malaria, HIV-AIDS, maternal and child health, adult and childhood mortality, women’s empowerment, domestic violence, and other health-related issues. Four questionnaires were used for the data collection: Household Questionnaire, Women’s Questionnaire, Men’s Questionnaire and Biomarker Questionnaire. The Women dataset which contains information on sexual and reproductive health of all women of childbearing age was used for our study. Further information about the 2016/17 HDHS is provided in the full report [29].

### Sample design

The 2016/17 HDHS followed a two-stage sample design and was intended to allow estimates of key indicators at the national level as well as for urban and rural areas and each of Haiti’s 10 administrative regions (Ouest, Sud, Sud-Est, Grande-Anse, Nippes, Nord, Nord-Ouest, Nord-Est, Centre, and Artibonite). In the first stage, 450 enumeration areas (EAs) or clusters were selected with probability proportional to sample enumeration area (SEA) size. The second stage involved a systematic selection of households from the selected EAs. In the 450 EAs, 13,451 households were occupied at the time of data collection of which 13,405 were successfully interviewed. Further, 14,525 women of childbearing age (15–49 years) were eligible to participate and 14,371 were successfully interviewed, yielding a response rate of 98.9%. The men’s survey was conducted in two-thirds of the sample households, and all men aged 15–64 who were either permanent residents of the selected households or visitors who stayed in the households the night before the survey in these households were included. Out of a total of 9995 men who were identified, 9795 were successfully interviewed, yielding a response rate of 98.0%. Finally, in one-third of the households, women aged 50–64 and men aged 35–64 were also eligible, but only for certain aspects of the survey. In this subsample, 2125 men aged 35–64 were identified and 2091 were successfully interviewed, yielding a response rate of 98.4%. As for women aged 50–64, 1150 were eligible, and 1142 were successfully interviewed (99.3% of response rate) [29].

### Data collection

The interviewers (recruited and trained by ICF Institutional Review Board, HIC, HNBS, and MPHP staffs), conducted face-to-face interviews in the selected households from eligible women and men. The data was collected via the Computer Assisted Personal Interviewing (CAPI) data collection system developed by the DHS Program. The tablet PCs were used to record the responses obtained [29].

### Study population

All women of childbearing age (15–49 years) successfully interviewed constitute our study population.

### Study variables and measurements

#### Outcome variable

The outcome variable of this study was having correct knowledge of ovulatory cycle (KOC), which was recoded and dichotomized. When collecting data from the women, they were asked “when do you think a woman has the greatest chance of becoming pregnant?”. The different responses were: “during her period", "after period ended", "middle of the cycle", "before period begins", "at any time", and "I don’t know". Respondents who indicated fertile period is at the middle of the menstrual cycle were categorized as having correct KOC while respondents who answered the question as fertile period is “during her period”, “after period ended”, “before period begins”, “at any time”, and “I don’t know” were categorized as “no”. This variable coding is provided by the HDHS [29].

#### Independent variables

The covariates were divided into three groups socio-demographic variables which included age (“less than 25 years”, “25–29”, “30–34”, “35–39”, “40 and above”), place of residence (“urban” and “rural”), region (“Aire Métropolitaine de Port-au-Prince”, “Reste-Ouest”, “Grand Sud”, “Grand Nord”, “Artibonite”, “Centre”, and “Grand’Anse/Nippes”), religion (“no religion”, “christian”, and “voodoo or others”), respondent's education level (“primary or less”, “secondary”, and “higher”), marital status (“never in union”, “in union”, and “widowed/divorced/separated”), number of living children (“no children”, “1–2”, “3 or more”), husband/partner’s education level (“primary or less”, “secondary”, and “higher”); socio-economic variables which included wealth index (“poor”, “middle”, and “rich”) and currently working (“yes” and “no”). The other independent variables were current contraceptive use by method type (“no method”, “traditional method”, and “modern method”), fieldworker visit in last 12 months (“yes” and “no”), and exposure to mass media family planning messages (“yes” and “no”). Exposure to mass media family planning messages was a composite variable created by combining four variables: “have heard about family planning messages on radio in last few months”, “have heard about family planning messages on TV in last few months”, “have heard about family planning in newspaper/magazine in last few months”, and “have heard about family planning by text messages on mobile phone in last few months”. In the *Women* dataset, these four variables were coded as “yes”, and “no”. After examining the frequency distribution of the responses, we coded it as “yes” if a woman heard family planning messages through at least one of these mass media, and “no” if she did not hear family planning messages through any of the mass media. Selection of these covariates was based on previous studies [1, 18–21].

### Statistical analysis

Descriptive statistics were generated to summarize the data and the results were presented as proportions (%). Bivariable analyses (Pearson’s chi-square) were conducted to assess the associations between the outcome variable and each covariate. To identify determinants of KOC, a multi-variable analysis (binary logistic regression) was performed and adjusted odds ratios (AOR) at 95% confidence intervals (95% CI) were estimated. All analyses were weighted (HV005/1,000,000) to get unbiased estimates [29]. Statistical analysis was done in STATA 14 software using *svy* command to adjust for the complex sampling structure of the data [30]. A significance level of 0.05 was considered.

### Ethical consideration

This study is based on a secondary analysis of publicly available data (https://dhsprogram.com/data/available-datasets.cfm); therefore, no ethics approval was required from our institutions. However, to obtain permission to access these datasets, we registered and sent a request (on May 3, 2022) including the objective of our study to the managers of the DHS program via their online platform. Two days later, we received approval to access and download the data files. According to the 2016/2017 HDHS report, during DHS data collection, all participants gave their informed consent, and the data were anonymized [29].

## Results

### Socio-economic and demographic characteristics of the study participants

Table 1 summarises the characteristics of the study participants. Slightly more than 40.0% (41.8%) of women were young (less than 25 years), 15.7% were aged 25–29 years, 14.0% were 30–34 years, 11.5% were in the 35–39-year age group, and 16.9% were aged 40 years and above. The vast majority (90.8%) were Christians; more than half (53.2%) were from rural areas and 25.3% came from “Aire Métropolitaine de Port-au-Prince” region. Around a third of them (32.0%) were in the poor wealth index category, 43.7% were not working, and 43.5% had primary education or less.

**Table 1:** Socio-economic and demographic profiles of women of childbearing age in Haiti

Socio-economic and demographic characteristics	All women of childbearing age	
	N	Percentage
*Age*		
Less than 25 years	6012	41.8
25–29	2258	15.7
30–34	2016	14.0
35–39	1650	11.5
40 and above	2435	16.9
*Place of residence*		
Urban	6731	46.8
Rural	7640	53.2
*Region*		
Aire Métropolitaine de Port-au-Prince	3632	25.3
Reste-Ouest	2285	15.9
Grand Sud	1708	11.9
Grand Nord	2778	19.3
Artibonite	2090	14.5
Centre	918	6.4
Grand'Anse/Nippes	959	6.7
*Wealth index*		
Poor	4596	32.0
Middle	2772	19.3
Rich	7003	48.7
*Currently working*		
Yes	8091	56.3
No	6280	43.7
*Religion*		
No religion	1134	7.9
Christian	13,055	90.8
Voodoo or others	182	1.3
*Education level*		
Primary or less	6258	43.5
Secondary	7068	49.2
Higher	1045	7.3
*Marital status*		
Never in union	5823	40.5
In union	7402	51.5
Widowed/divorced/separated	1146	8.0
*Number of living children*		
No children	5990	41.7
1–2	4586	31.9
3 or more	3795	26.4
*Husband/partner's education level*		
Primary or less	3558	24.8
Secondary	3051	21.2
Higher	693	4.8
Don’t know	101	0.7
No partner	6969	48.5
*Knowledge of any contraceptive method*		
Knows modern method	14,351	99.9
Knows no method	20	0.1
*Current use by method type*		
No method	10,909	75.9
Traditional method	263	1.8
Modern method	3199	22.3
*Exposure to FP messages (medias)*		
Yes	3929	27.3
No	10,442	72.7
*Visited by fieldworker in last 12 months*		
Yes	991	6.9
No	13,380	93.1
Total	14,371	100.0

Over half (51.5%) of the women were in union, and about 60.0% had at least one child with 26.4% having 3 or more children. Almost all of the participants had knowledge of modern contraceptive methods whilst only 22.3% reported using a modern method during sexual intercourse. Nearly a quarter (24.8%) declared that their partner had primary education or less, 26.0% reported that their partner had secondary level or higher, and 48.5% didn’t have partner. Less than 30.0% of them had been exposed to mass media family planning (FP) messages in last few months preceding the survey, and 6.9% had been visited by a fieldworker.

### Association between socio-economic and demographic characteristics and KOC

Table 2 depicts the bivariable associations between KOC and socio-economic and demographic characteristics. Overall, the proportion of women who reported having correct knowledge of ovulatory cycle was 24.1% (95% CI = 23.4–24.8). The results indicated that participants’ responses on KOC varied markedly. The lowest proportion of correct KOC was among women aged 40 and above (19.1%), while the highest proportions were among those in the 25–29 (27.6%) and 30–34 (27.8%) age groups. Approximately 30.0% of urban women were knowledgeable about ovulatory cycle compared to 19.7% in rural areas. Correct KOC was most common in “Aire Métropolitaine de Port-au-Prince” region (30.2%) and least common in “Centre” (19.0%). Similarly, correct KOC prevalence was higher among women who had higher education level (52.7%), from rich households (30.3%) and currently working (24.8%).

**Table 2:** Bivariable association between KOC and socio-economic and demographic characteristics

Socio-economic and demographic characteristics	Knowledge of ovulatory cycle		*p*-value
	Yes (N/%)	No (N/%)	
*Age*			0.000
Less than 25 years	1383 (23.0)	4629 (77.0)	
25–29	624 (27.6)	1634 (72.4)	
30–34	560 (27.8)	1456 (72.2)	
35–39	426 (25.8)	1224 (74.2)	
40 and above	466 (19.1)	1969 (80.9)	
*Place of residence*			0.000
Urban	1950 (29.0)	4781 (71.0)	
Rural	1509 (19.7)	6131 (80.3)	
*Region*			0.000
Aire Métropolitaine de Port-au-Prince	1097 (30.2)	2535 (69.8)	
Reste-Ouest	498 (21.8)	1787 (78.2)	
Grand Sud	420 (24.5)	1288 (75.5)	
Grand Nord	641 (23.1)	2137 (76.9)	
Artibonite	425 (20.3)	1665 (79.7)	
Centre	174 (19.0)	744 (81.0)	
Grand'Anse/Nippes	204 (21.3)	755 (78.7)	
*Wealth index*			0.000
Poor	737 (16.0)	3859 (84.0)	
Middle	601 (21.7)	2171 (78.3)	
Rich	2121 (30.3)	4882 (69.7)	
*Currently working*			0.014
Yes	2010 (24.8)	6081 (75.2)	
No	1449 (23.1)	4831 (76.9)	
*Religion*			0.000
No religion	175 (15.4)	959 (84.6)	
Christian	3245 (24.9)	9810 (75.1)	
Voodoo or others	39 (21.3)	143 (78.7)	
*Education level*			0.000
Primary or less	933 (14.9)	5325 (85.1)	
Secondary	1975 (27.9)	5093 (72.1)	
Higher	551 (52.7)	494 (47.3)	
*Marital status*			0.000
Never in union	1522 (26.1)	4301 (73.9)	
In union	1682 (22.7)	5720 (77.3)	
Widowed/divorced/separated	255 (22.3)	891 (77.7)	
*Number of living children*			0.000
No children	1608 (26.8)	4382 (73.2)	
1–2	1139 (24.8)	3447 (75.2)	
3 or more	712 (18.7)	3083 (81.3)	
*Husband/partner's education level*			0.000
Primary or less	573 (16.1)	2985 (83.9)	
Secondary	799 (26.2)	2251 (73.8)	
Higher	291 (42.0)	402 (58.0)	
Don't know	19 (19.0)	81 (81.0)	
No partner	1777 (25.5)	5193 (74.5)	
*Current use by method type*			0.000
No method	2507 (23.0)	8402 (77.0)	
Traditional method	94 (35.7)	169 (64.3)	
Modern method	858 (26.8)	2341 (73.2)	
*Exposure to FP messages (medias)*			0.000
Yes	1314 (33.4)	2615 (66.6)	
No	2145 (20.5)	8297 (79.5)	
*Visited by fieldworker in last 12 months*			0.000
Yes	303 (30.6)	688 (69.4)	
No	3156 (23.6)	10,224 (76.4)	
Total	3459 (24.1)	10,912 (75.9)	

Nearly a quarter (24.9%) of Christian respondents had correct KOC and this proportion was 15.4% and 21.3% among non-religious women and voodoo adherents, respectively. The results also showed that women who were not in union (26.1%) had a higher prevalence of correct KOC than those who were in union (22.7%) or widowed/divorced/separated (22.3%). Women with no children had a prevalence of correct KOC of 26.8%, while those who had 3 or more children had a prevalence of 18.7%. Slightly more than 40.0% of women whose partners had higher education level reported having correct KOC compared to 16.1% among those whose partners had primary education or less. In addition, correct KOC was most common among respondents who used traditional contraceptive methods (35.7%) and least common among those who declared using no method (23.0%). As expected, correct KOC prevalence was higher among women who had been exposed to mass media FP messages (33.4%) and visited by a fieldworker (30.6%) than their counterparts who had not been exposed to mass media FP messages (20.5%) and not been visited by a fieldworker (23.6%). In summary, it should be noted that a statistically significant association was found between all selected covariates and correct KOC (*p* < 0.05).

### Factors associated with correct KOC

In the binary logistic regression, place of residence, respondent’s education level, wealth index, currently working, husband/partner’s education level, contraceptive use, exposure to mass media FP messages, and fieldworker visit were statistically significant predictors of correct KOC at *p*-value < 0.05 (Table 3). Urban women had higher odds (AOR = 1.10; 95% CI 1.01–1.21) of having correct KOC compared to rural women. The odds of correct KOC were higher among women who attended secondary (AOR = 1.95; 95% CI 1.75–2.17) and higher level (AOR = 4.58; 95% CI 3.81–5.49) compared to those with primary level or less. Similarly, participants whose partners had secondary (AOR = 1.20; 95% CI 1.05–1.37) and higher education (AOR = 1.30; 95% CI 1.05–1.62) were more likely to have correct KOC than their counterparts with primary education or less. We also found that women from rich households were 1.2 times more likely to have correct KOC (AOR = 1.24; 95% CI 1.14–1.35) compared to those from poor households. Participants who currently worked had 1.1 greater odds (AOR = 1.13; 95% CI 1.03–1.25) of having correct KOC than those who were not currently working. Participants who reported using no contraceptive method (AOR = 0.82; 95% CI 0.74–0.90) had lower odds of having correct KOC compared with those who declared using modern method. In addition, women who had been exposed to mass media FP messages had 1.7 greater odds (AOR = 1.67; 95% CI 1.53–1.83) of having correct KOC than their counterparts who had not been exposed to mass media FP messages. Likewise, women who had been visited by a fieldworker were 1.5 times more likely (AOR = 1.51; 95% CI 1.30–1.75) to have correct KOC than those who had not been visited.

**Table 3:** Multi-variable association (binary logistic regression) between KOC and socio-economic and demographic characteristics

Socio-economic and demographic variables	Coef	Adjusted odds ratio (AOR)	95% CI
*Age*			
Less than 25 years	− 0.0565	0.95	0.80–1.12
25–29	− 0.0274	0.97	0.83–1.14
30–34	0.0409	1.04	0.90–1.22
35–39	0.1021	1.11	0.94–1.30
Ref = 40 and above			
*Place of residence*			
Urban	0.0997	1.10*	1.01–1.21
Ref = Rural			
*Region*			
Reste-Ouest	0.0977	1.10	0.94–1.29
Grand Sud	0.1261	1.13	0.99–1.30
Grand Nord	0.0126	1.01	0.91–1.13
Artibonite	− 0.0429	0.96	0.83–1.11
Centre	− 0.0884	0.92	0.77–1.10
Grand'Anse/Nippes	− 0.0841	0.92	0.81–1.04
Ref = Aire Métropolitaine de Port-au-Prince			
*Education level*			
Secondary	0.6669	1.95***	1.75–2.17
Higher	1.5208	4.58***	3.81–5.49
Ref = Primary or less			
*Wealth index*			
Middle	0.0702	1.07	0.97–1.19
Rich	0.2158	1.24***	1.14–1.35
Ref = Poor			
*Currently working*			
Yes	0.1252	1.13**	1.03–1.25
Ref = No			
*Religion*			
No religion	− 0.1472	0.86	0.72–1.04
Vodoo or others	0.0851	1.09	0.72–1.66
Ref = Christian			
*Marital status*			
In union	− 0.0921	0.91	0.76–1.10
Widowed/divorced/separated	0.0695	1.07	0.86–1.33
Ref = Never in union			
Number of living children			
No children	0.0712	1.07	0.89–1.30
1–2	0.0092	1.01	0.89–1.15
Ref = 3 or more			
*Husband/partner's education level*			
Secondary	0.1813	1.20**	1.05–1.37
Higher	0.2639	1.30**	1.05–1.62
Don't know	− 0.2780	0.7573	0.40–1.42
Ref = Primary or less			
*Current use by method type*			
No method	− 0.2027	0.82***	0.74–0.90
Traditional method	0.1703	1.19	0.89–1.59
Ref = Modern method			
*Exposure to FP messages (medias)*			
Yes	0.5141	1.67***	1.53–1.83
Ref = No			
*Visited by fieldworker in last 12 months*			
Yes	0.4114	1.51***	1.30–1.75
Ref = No			

^*^p < .05; **p < .01; ***p < .001

## Discussion

The aim of this study was to assess the level of correct KOC and its determinants among women of childbearing age in Haiti, using the 2016/17 HDHS. Several significant findings are revealed in this study. First, we found that the overall proportion of women of childbearing age in Haiti having correct KOC was estimated at 24.1% (95% CI 23.4–24.8), two times higher than in the Dominican Republic (12.0%) [31]. This could be partly explained by the fact that women in the Dominican Republic have more access to modern FP information and services, and use natural FP methods less than Haitian women [32, 33]. Second, place of residence, respondent’s education level, wealth index, currently working, husband/partner’s education level, contraceptive use, exposure to mass media FP messages, and fieldworker visit were identified as determinants of correct KOC.

This study found that education level was positively and significantly associated factor with correct KOC. Women with higher education status were more likely to have correct KOC compared to those with primary education or no education. Similarly, higher education level of husband/partner contributes to women’s correct KOC. Consistent with findings from previous studies [16, 34, 35], this could be explained by the fact that individuals with higher education level are literate and better informed on topics related to reproductive health [36, 37]. Moreover, when a husband/partner has higher education, they are more open to discussions about sexual health with their partners, which could enhance the level of women's knowledge of the ovulatory cycle [32, 33].

Urban women were more knowledgeable than rural women, which is supported by prior studies [18, 38, 39]. This might be due to a variety of reasons. Urban women in Haiti have greater access to sexual and reproductive health information and services [40, 41]. Besides, they are better educated than their rural counterparts [29], which would allow them to better comprehend information about the ovulatory cycle [7].

It was also revealed that exposure to mass media FP messages significantly predicted correct KOC. Women who had been exposed to mass media FP messages had greater odds of having correct KOC than women who had not been exposed. Wolde et al. [20] reported similar findings in Ethiopia. During the last decades, the mass media has been considered as a powerful tool for communication [36]. Additionally, studies have shown that mass media remains a vital source of sexual information and can raise awareness, increase knowledge, and influence individual behaviours and attitudes [36, 42]. This observation underscores the importance for the Haitian government to provide electricity to more households in Haiti in order to allow these messages to reach more women, especially those in rural areas. Further, consistent with findings from studies in Bangladesh [22] and Ethiopia [32], we found that being visited by a fieldworker increases the likelihood of correct KOC. The possible explanation for this finding is that women who had been visited by fieldworkers had the possibility to have adequate information on ovulatory cycle, and inquire about their specific sexual and reproductive health issues.

Wealth index and currently working were other significantly associated factors with women’s correct KOC. The odds of having correct KOC was higher among women from rich households and those who were currently working compared to women from poor households and without income-generating activities, respectively. These results are in line with several studies [7, 18, 20, 22, 32, 43] that suggested that women from wealthy households and working women are more likely to afford sexual health services compared with their counterparts from poor households and without income-generating activities. Furthermore, our data indicated that women from rich households and currently working were in significantly higher proportions, living in urban areas, with a higher education level, exposed to mass media FP messages, and receiving visit by a fieldworker than those from poor households and without income-generating activities, respectively, which could positively influence their correct KOC level.

In agreement with a recent study in Malawi [44], our results indicated that women who used modern contraceptive methods were more likely to have correct KOC than those who used no method. It is possible that women who use modern contraception have knowledge of their ovulatory cycle and use modern contraception to avoid unintended pregnancy [40, 41].

### Study strengths and limitations

To the best of the authors’ knowledge, this is the first study to assess the level of correct KOC and its determinants among women of childbearing of age in Haiti using the 2016/17 HDHS (nationally representative DHS data). Further, the findings are based on adequate statistical power (data were weighted for the sampling probabilities) and took into account the complex sampling procedures in the analysis. Although this study contributes to the literature on knowledge of ovulatory cycle, it has some limitations. First, the study used exclusively a quantitative research design. Future studies using qualitative methods will provide a more nuanced understanding of the factors associated with correct KOC. Second, due to the cross-sectional study design, we could not infer causality in the relationships between the exposure variables and the outcome. Third, due to the self-reported nature of the DHS surveys, the data may be subject to recall bias. Finally, failure to control for known and unknown potential confounding factors due to data limitations could have resulted in an over- or under-estimation of the associations between the socioeconomic and demographic variables assessed with KOC.

### Implications of the findings

Knowledge of ovulatory cycle is an important factor in fertility awareness and decision-making [32]. This is especially important in the context of Haiti, characterized by low contraceptive prevalence and high unmet need for modern contraception [24, 45]. Promoting correct KOC through education and media outreach may be helpful strategies for enhancing fertility decision-making and, subsequently, increasing contraceptive use, including modern methods, as well as fertility-based methods such as natural family planning methods, which depend on accurate KOC.

## Conclusion

Prevalence of correct knowledge of ovulatory cycle (KOC) is low among women of childbearing age in Haiti. Women from rural and poor backgrounds, without income-generating activities, with lower education, not using contraception, not exposed to mass media FP messages, not visited by fieldworker and with less educated spouses/partners lack the adequate knowledge of ovulatory cycle. It should also be noted that there is a deep and long-lasting lack of public investment in education in Haiti. Despite of a significant growth in the number of children having access to primary/secondary school, in the last decades, the quality has not grown the same. There are a lot of unskilled persons teaching. This could also partly explain low prevalence of correct KOC. Thus, priority should be accorded to increasing the knowledge of ovulatory cycle among childbearing age women, especially women with unmet need for contraception (who are neither using traditional nor modern contraception methods). These findings showed that policies should be developed to increase the knowledge about women’s menstrual and ovulatory cycle, especially for women from at risk demographic groups.

## Data Availability

The dataset used in this study is available on the following repository: https://dhsprogram.com/data/dataset/Haiti_Standard-DHS_2016.cfm?flag=0.
